# Metatranscriptomic analysis of common mosquito vector species in the Canadian Prairies

**DOI:** 10.1128/msphere.00203-24

**Published:** 2024-06-24

**Authors:** Cole Baril, Bryan J. Cassone

**Affiliations:** 1 Department of Biology, Brandon University, Brandon, Manitoba, Canada; University of Pittsburgh, Pittsburgh, Pennsylvania, USA

**Keywords:** *Aedes*, *Ochlerotatus*, *Culex*, viruses, pathogens, next-generation sequencing

## Abstract

The microbiome plays vital roles in the life history of mosquitoes, including their development, immunity, longevity, and vector competence. Recent advances in sequencing technologies have allowed for detailed exploration into the diverse microorganisms harbored by these medically important insects. Although these meta-studies have cataloged the microbiomes of mosquitoes in several continents, much of the information currently available for North America is limited to the state of California. In this study, we collected >35,000 mosquitoes throughout Manitoba, Canada, over a 3-year period and then harnessed RNA sequencing and targeted reverse transcriptase-PCR to characterize the microbiomes of the eight most pervasive and important vector and pest species. The consensus microbiome of each species was overwhelmingly composed of viruses but also included fungi, bacteria, protozoa, and parasitic invertebrates. The microbial assemblages were heterogeneous between species, even within the same genus. We detected notable pathogens, including the causal agents of Cache Valley Fever, avian malaria, and canine heartworm. The remaining microbiome consisted largely of putatively insect-specific viruses that are not well characterized, including 17 newly discovered viruses from 10 different families. Future research should focus on evaluating the potential application of these viruses in biocontrol, as biomarkers, and/or in disrupting mosquito vectorial capacity. Interestingly, we also detected viruses that naturally infect honeybees and thrips, which were presumably acquired indirectly through nectar foraging behaviors. Overall, we provide the first comprehensive catalog of the microorganisms harbored by the most common and important mosquito vectors and pests in the Canadian Prairies.

**IMPORTANCE:**

Mosquitoes are the most dangerous animals on the planet, responsible for over 800,000 deaths per year globally. This is because they carry and transmit a plethora of human disease-causing microorganisms, such as West Nile virus and the malaria parasite. Recent innovations in nucleic acid sequencing technologies have enabled researchers unparalleled opportunities to characterize the suite of microorganisms harbored by different mosquito species, including the causal agents of disease. In our study, we carried out 3 years of intensive mosquito surveillance in Canada. We collected and characterized the microorganisms harbored by >35,000 mosquitoes, including the identification of the agents of Cache Valley fever, avian malaria, and canine heartworm. We also detected insect-specific viruses and discovered 17 new viruses that have never been reported. This study, which is the first of its kind in Canada and one of only a handful globally, will greatly aid in future infectious disease research.

## INTRODUCTION

Microbiome refers to the assemblage of microorganisms harbored by a given organism, including viruses, bacteria, protozoans, and fungi (1, 2). A range of biotic and abiotic factors can have a profound effect on the microbiome, which in turn may impact the life history of the host. For mosquitoes, their microbiome plays critical roles in development, longevity, immunity, and vector competence (2–8). Relatively recent advances in sequencing technologies have greatly expanded our understanding of the highly diverse and dynamic microbial flora carried by mosquitoes (9–13). These studies indicate that the microbiome can vary among different mosquito species, within populations of the same species, and even among individual mosquitoes within populations. Consequently, cataloging the microbial communities of notable mosquito vector species within a given geographical region is likely to provide key insights into their life history traits, with potential applications for disease and pest control.

A major component of the mosquito microbiome are viruses (i.e., the virome), some of which (i.e., arboviruses) may be transmitted to humans, livestock, and other animals (14–16). Of the ~3,500 extant mosquito species, only a small proportion of vector viruses are of public health or veterinary importance, with the majority belonging to the genera *Aedes* and *Culex* (17). In the Canadian Prairies, the most ubiquitous mosquito vector is the inland floodwater mosquito, *Aedes vexans* Meigen (18). This species is capable of transmitting West Nile virus (WNV), California serogroup viruses (CSGVs), Zika virus, and Rift Valley fever virus (19–22). The summer saltmarsh mosquito, *Ochlerotatus dorsalis* Meigen, and *Culex tarsalis* Coquillett are also commonly found species in the Prairies. Both are competent vectors of Western equine encephalitis virus (WEEV), WNV, and CSGVs (23, 24). The cattail mosquito, *Coquillettidia perturbans* Walker, is distributed across the Prairies and typically breeds in permanent swamps where cattails and other aquatic plants are present (23). In addition to WNV and CSGVs, this mosquito is a carrier of Eastern equine encephalitis virus (EEEV) (23, 25). Other mosquito vector species occurring in the Prairies include *Aedes canadensis* Theobald (CSGVs, WNV, and EEEV), *Ochlerotatus triseriatus* Say (La Crosse virus, EEEV, and WEEV), *Ochlerotatus flavescens* Müller (canine heartworm, *Dirofilaria immitis*) (23, 24, 26–28). It should be noted that *Ochlerotatus* was previously ranked as a subgenus of *Aedes* but has since been reclassified as a distinct genus (29).

In addition to arboviruses, the mosquito virome includes insect-specific viruses (ISVs) (30). As their name suggests, these viruses establish an infection in mosquitoes and/or other insects but are incapable of replicating in vertebrate hosts. These viruses are thought to be vertically transmitted transovarially by mosquitoes from infected females to their offspring (31–33), though the mechanisms by which ISVs establish an infection in the mosquito is not fully known (34). Insect-specific viruses have been recognized for decades (35) but were vastly understudied until recent advancements in microbiome research, sequencing technologies, bioinformatics tools, and phylogenetic analyses. Indeed, the genomes or partial genomes of hundreds of ISVs from diverse viral families (e.g., *Bunyaviridae, Flaviviridae, Reoviridae, Rhabdoviridae, Togaviridae, Birnaviridae, Nodaviridae, Phenuiviridae,* and *Mesoniviridae*) have now been sequenced and characterized from various geographical locations [for review, see references (36, 37)]. Despite their strict host tropism with no (known) direct implications to the burden of infectious diseases, ISVs may impact/regulate mosquito vector competence (34, 38–40) and in some cases may be used in biocontrol (41) or serve as effective biomarkers for viruses of public health concern (42, 43).

Two Canadian Prairie provinces (Manitoba and Saskatchewan) carry out mosquito surveillance activities annually to identify and assess the prevalence of arboviruses carried by vector species. However, these provincial programs are mostly limited to monitoring *Cx. tarsalis* for WNV infection using serological and/or reverse transcriptase-PCR (RT-PCR)-based methodologies. Consequently, there may be other arboviruses contributing to an under-recognized burden of disease within this region. For instance, clinical cases of CSGVs have been reported in Manitoba (44, 45), and mosquitoes harboring the viruses have been detected in the adjacent US state, North Dakota (24). Furthermore, nothing is known regarding the ISVs or other microorganisms (e.g., protozoa, fungi, and bacteria) that may contribute to the microbiome of common vector species in the Canadian Prairies. Metatranscriptomics has filled in these knowledge gaps for mosquito species in other geographical regions, including studies from Asia (46), Australia (47, 48), USA (12, 49, 50), and the Caribbean (9). With this in mind, we carried out RNA sequencing in over 35,000 mosquitoes collected throughout Manitoba, Canada, over a 3-year period. The microbiomes of eight of the most common vector and pest species were characterized, which included an array of viruses (arboviruses, ISVs, and novel viruses), as well as bacteria, fungi, protozoa, and invertebrate parasites. We also carried out targeted RT-PCR-based diagnostics to assess the prevalence of CSGVs in the competent vector species.

## MATERIALS AND METHODS

### Mosquito collections and identification

**Fig 1:**
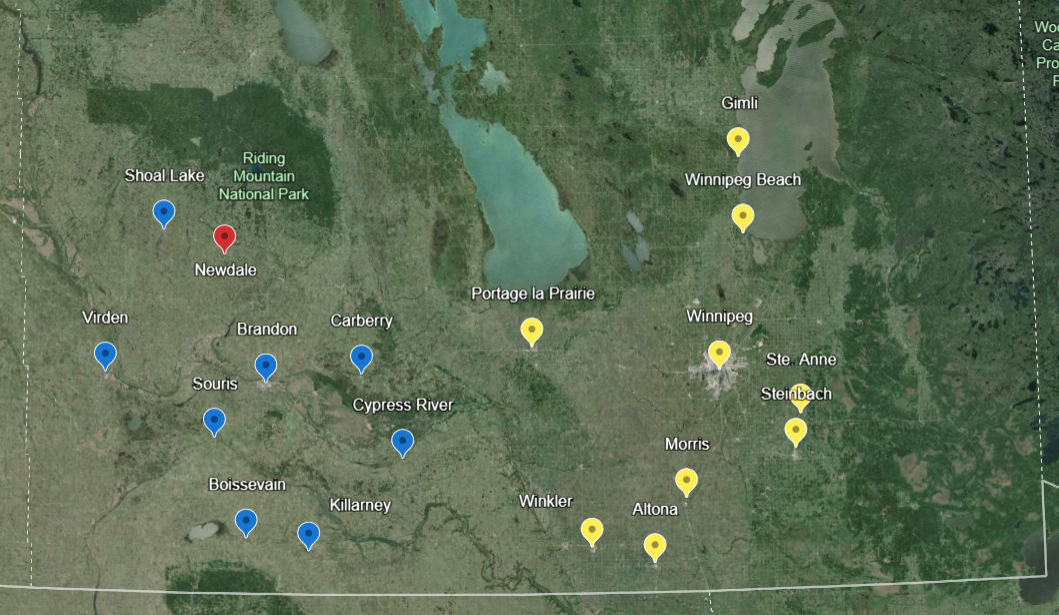
Sampling sites throughout southern Manitoba. Mosquitoes were captured on a weekly batsis in western sites (blue) between June and September, whereas one-time satellite collections were carried out at various times between June and September for eastern sites (yellow). A one-off trapping site (red) was also included (pool 41). The map used in the figure was generated in Google Earth (version 7.3).

Host-seeking females were trapped between June and August in 2020 and 2021, as previously described (18). In brief, CDC Miniature Light Traps (Model 1012; John W. Hock, Gainesville, FL, USA) were placed on tree limbs ~1.5 m from the ground with carbon dioxide regulators set to 15 psi and the light disabled. Traps were activated from dusk until dawn twice weekly (Monday and Tuesday) in eight West Manitoba communities. In 2020, the City of Winnipeg Insect Control Branch provided us with one-time satellite traps from nine additional locations in Central and East Manitoba. In 2019, weekly collections were also done at four sites located in Brandon, Manitoba, between July and August. Figure 1 shows the sampling localities throughout the province, whereas Table S1 provides a brief description of each site.

Mosquito vector species were visually identified using dissecting microscopes and applicable identification keys (23, 51, 52). Eight target species were sorted out with the remaining specimens omitted from our study: *Aedes vexans* Meigen, *Aedes canadensis* Theobald, *Ochlerotatus dorsalis* Meigen, *Ochlerotatus flavescens* Muller, *Culex tarsalis* Coquillett, *Coquillettidia perturbans* Walker, and *Anopheles earlei* Vargus. We also targeted *Ochlerotatus triseriatus* Say, which was distinguished from the closely related *Ochlerotatus hendersoni* via molecular screening using the approach described in reference (28). Mosquitoes were then pooled into 1.5-mL tubes containing up to 50 individuals sorted by species, location, and year. Pools were stored at −80°C until molecular analysis.

### RNA isolation and sequencing

Total RNA was extracted from each pooled mosquito sample using the RNeasy Mini Kit (Qiagen, Hilden, Germany), following the manufacturer’s recommended protocol. The quantity, quality, and concentration of RNA were evaluated using a Nanophotometer NP80 (Implen Inc., Westlake Village, CA, USA). We then combined RNA samples derived from the same species, location, and year to form larger pools for sequencing (Table S2). Most sequencing pools reflect specimens caught from June to September of a given year. In instances where a pool had relatively few specimens, we combined mosquitoes from neighboring sites or collection years for a given species. Approximately 2 µg of RNA per pool sample was sent to Génome Québec Innovation Centre (McGill University, Montreal, QC, Canada) for mRNA library preparation (New England Biolabs, Ipswich, MA, USA) and paired read sequencing (100 bp) on the NovaSeq platform (Illumina, San Diego, CA, USA). We then used CASAVA 1.8.2 to carry out bcl conversions and demultiplexing. Image deconvolution and quality value calculations were performed using the Illumina GA pipeline (version 1.6). The raw sequence reads can be retrieved from the National Center for Biotechnology Information (NCBI) Short Sequence Read Archive (SRA) under the (SRA) accession number PRJNA793247.

### Read processing and *de novo* assembly

The Chan Zuckerberg ID Metagenomic mNGS Pipeline (version 6.8; Chan Zuckerberg Biohub, CZID), an open-sourced cloud-based bioinformatics platform (https://czid.org/), was used for quality control and host filtration of reads, as described by references (12) and (53). Each sample (i.e., mosquito pool) was analyzed individually, as described below. The CZID pipeline employs STAR and Bowtie2 to perform host filtration (human and mosquito), Trimmomatic for adapter trimming, Price Seq for removal of low-quality reads, LZW for the removal of low complexity reads, and CZIDdedup for duplicate read identification.

Preprocessed host filtered reads were then downloaded from CZD and assembled *de novo* into contig sequences using CLC Genomics workbench (version 20; CLC Bio, Aarhus, Denmark) with the following parameters: mismatch cost = 2, insertion cost = 3, deletion cost = 3, length fraction = 0.7, and similarity fraction = 0.95 (default settings therein).

### Microorganism identification

To identify sequences of microbial origin, contigs were subjected to desktop-downloaded BLASTn and tBLASTx searches against the NCBI nucleotide (nt) and non-redundant databases, respectively. Each contig was also mapped against the local NCBI virus blast (curated via Entrez Query in May 2023) using CLC and a customized database omitting improbable viruses (e.g., HIV and influenza). We used the following criterion to categorize novel and non-novel viruses: minimum *E* value of ≤1 × 10^−100^, nucleotide and amino acid similarities of >90%, and contig length of ≥250 nt. In addition, a minimum coverage of 10× was used as the cut-off value for a confirmed virus. Contigs meeting these criteria were further scrutinized through analysis of protein function using the NCBI ORFfinder and NCBI conserved domains tools to eliminate possible false positives. Contigs of viral origin with percent amino acid identities <85% were flagged as potentially novel viruses.

BLAST results were passed through a custom pipeline to filter and bin results based on our criteria built using R (version 4.2.2). Data manipulation was carried out using a combination of purrr (version 1.0.1) (54), dplyr (version 1.1.2) (55), tidyr (version 1.3.0) (56), janitor (version 2.2.0) (57), and phylotools (version 0.2.2) (58). The assertr (version 3.0.0) (59) package was used to validate the data and implement quality control checks. Tables were generated using the gt (version 0.9.0) (60) package. Figures were generated using the ggplot2 (version 3.4.2) (61), ggVennDiagram (version 1.2.2) (62), and countrycode (version 1.4.0) (63) packages. NCBI metadata for each accession number was obtained using the httr (version 1.4.6) (64) and jsonlite (version 1.8.7) (65) packages.

### Targeted screening for California serogroup viruses

In addition to the high-throughput analyses, we screened a subset of samples for CSGVs using RT-PCR. Reverse transcription was first performed on RNA pools using the RevertAid Kit (Thermo Fisher Scientific Inc., Waltham, MA, USA), following the manufacturer recommended protocol. Amplification of a 251-nt fragment of the S segment was then carried out using Platinum SuperFi PCR Master Mix (Thermo Fisher Scientific Inc.) and the following primer set: BCS82C: 5′-ATGACTGAGTTGGAGTTTCATGATGTCGC-3′ and BCS332V: 5′-TGTTCCTGTTGCCA GGAAAAT-3′ (66). Thermocycler (Biometra TOne, Analytics Jena, Germany) conditions consisted of 39 cycles of 94°C for 1 min, 56°C for 1 min, and 72°C for 1 min, with a final extension at 72°C for 5 min. Amplicons were visualized in 1% agarose gels stained with ethidium bromide using a ChemiDoc Imaging System (Bio-Rad Laboratories, Hercules, CA, USA). We then sent amplicons to the Génome Québec Innovation Centre (McGill University) for purification using a Biomek NX robot with a bead solution and Sanger sequencing of the forward strand using the 3730xl DNA Analyzer (Applied Biosystems, Waltham, MA, USA). Sequences were identified as CSGVs using BLASTn and tBLASTx.

## RESULTS

### Mosquito microbiomes are largely composed of viruses

A total of 45 cDNA libraries representing 35,866 mosquitoes collected throughout Manitoba, Canada, were subjected to RNA sequencing. The number of sequencing libraries and specimens sequenced per species varied considerably and largely reflected their prevalence in our sampling region. We generated more than 3.1 billion paired-end reads of 100 bp; Fig. S1 displays the number of reads passing quality control and host filtering for each library. It should be noted that the pooled sample sizes and number of reads per sample varied, which would impact the coverage for both the host and non-host organisms. The non-mosquito read subset of each cDNA library was then assembled *de novo* into contigs, functionally annotated via ortholog prediction, and classified to species level (where possible). Our results show an array of different types of organisms associated with the mosquito microbiome, with viruses comprising >99% (*n* = 5,637,086) of non-host reads mapping to contigs. Our finding of viruses making up the bulk of non-host reads is consistent with other metatranscriptomic studies (12). A total of 49 previously reported (i.e., known) viruses were identified in our data set, which included five types of viral genomes (+ssRNA, −ssRNA, dsDNA, dsRNA, and ssDNA) from 18 families (Table 1). The number of contigs assembled per virus ranged from 1 to 183, with a maximum contig length of 11.7 kb and a maximum number of reads of nearly 3 million (Table S3). Iflaviridae (*n* = 11) was the most represented viral family, followed by Rhabdoviridae (*n* = 8), Negevirus (*n* = 5), and Parvoviridae (*n* = 4). Nearly half (*n* = 23) of the viruses were detected in all three years (2019–2021).

**TABLE 1:** Classification, year(s) reported, and mosquito species in which each previously reported virus was recovered

Viral family	Virus^*a*^^*,^b^*^	Year(s)	Species (% sequencing pools present)^*c*^
Dicistroviridae	Black queen cell virus^*b*^	2019, 2020	*Ae. vexans* (5.3)
	Soybean thrips dicistrovirus^*b*^	2019–2021	*Ae. vexans* (15.8), *Cx. tarsalis* (45.5), and *Oc. dorsalis* (20)
Flaviviridae	Inari jingmenvirus	2020	*Ae. vexans* (5.3)
	Placeda virus^*a*^	2019–2021	*Ae. vexans* (10.5) and *Cx. tarsalis* (100)
Iflaviridae	Hanko iflavirus 1	2020, 2021	*Ae. vexans* (21.1), *Cq. perturbans* (16.7), *Cx. tarsalis* (9.1), *Oc. dorsalis* (100), and *Oc. flavescens* (100)
	Hanko iflavirus 2	2019–2021	*Ae. vexans* (57.9), *Cq. perturbans* (16.7), *Oc. dorsalis* (20)
	Hubei arthropod virus 1	2021	*Ae. vexans* (5.3)
	Pedersore iflavirus	2019–2021	*Ae. vexans* (36.8), *Cq. perturbans* (33.3), *Cx. tarsalis* (27.3), *Oc. dorsalis* (67), and *Oc. flavescens* (100)
	Thrace picorna-like virus 1	2021	*Ae. vexans* (10.5)
	Yongsan picorna-like virus 1	2019–2021	*Ae. vexans* (94.7) and *Oc. dorsalis* (60)
	Cafluga virus^*a*^	2020	*Cq. perturbans* (16.7) and *Oc. dorsalis* (20)
	Soybean thrips iflavirus 4^*b*^	2020	*Cq. perturbans* (16.7)
	Culex *i*flavi-like virus 4^*a*^	2019–2021	*Cx. tarsalis* (63.6)
	Culex iflavi-like virus 3^*a*^	2019–2021	*Cx. tarsalis* (100)
	Yongsan picorna-like virus 2	2019–2021	*Cx. tarsalis* (27.3)
Luteoviridae	Marma virus^*a*^	2019–2021	*Cq. perturbans* (16.7) and *Cx. tarsalis* (100)
Narnaviridae	Culex narnavirus 1^*a*^	2020	*Cx. tarsalis* (9.1)
Negevirus	Bro virus	2021	*Ae. canadensis* (100)
	Big cypress virus^*a*^	2020, 2021	*Ae. vexans* (15.8)
	Cordoba virus	2021	*Ae. vexans* (15.8)
	Mekrijarvi negevirus	2020, 2021	*Ae. vexans* (15.8), *Oc. dorsalis* (60)
	Utsjoki negevirus 3	2020	*Ae. vexans* (10.5)
Nodaviridae	Hubei noda-like virus 12	2021	*Cq. perturbans* (16.7) and *Cx. tarsalis* (9.1)
Tombusviridae	Des Moines River virus^*a*^	2019, 2020	*Ae. vexans* (5.3) and *Cx. tarsalis* (27.3)
	Hubei mosquito virus 4^*a*^	2019–2021	*Cx. tarsalis* (54.6)
	Tiger mosquito bi-segmented tombus-like virus	2021	*Cx. tarsalis* (9.1)
Tymoviridae	Hubei macula-like virus 3^*b*^	2019–2021	*Ae. vexans* (21.1) and *Oc. dorsalis* (20)
Virgaviridae	Hubei virga-like virus 2^*a*^	2019–2021	*Cx. tarsalis* (36.4)
Chuviridae	Chuvirus^*a*^	2019–2021	*Ae. vexans* (89.5), *Cx. tarsalis* (9.1), and *Oc. dorsalis* (60)
Orthomyxoviridae	Wuhan mosquito virus 6^*a*^	2019–2021	*Ae. vexans* (10.5), *Cq. perturbans* (16.7), and *Cx. tarsalis* (100)
	Astopletus virus^*a*^	2019–2021	*Cx. tarsalis* (100)
Peribunyaviridae	Culex bunyavirus 2^*a*^	2019–2021	*Cx. tarsalis* (100)
Rhabdoviridae	Flanders hapavirus^*a*^	2020, 2021	*Ae. vexans* (15.8) and *Cx. tarsalis* (90.9)
	Riverside virus 1^*a*^	2019–2021	*Ae. vexans* (73.7) and *Oc. dorsalis* (20)
	Canya virus^*a*^	2019–2021	*Cx. tarsalis* (63.6)
	Culex rhabdo-like virus	2020, 2021	*Cx. tarsalis* (27.3)
	Culex rhabdovirus^*a*^	2021	*Cx. tarsalis* (18.2)
	Elisy virus^*a*^	2019–2021	*Cx. tarsalis* (45.5)
	Manitoba virus^*a*^	2021	*Cx. tarsalis* (9.1)
	Merida virus^*a*^	2019–2021	*Cx. tarsalis* (100)
Birnaviridae	Ballard Lake virus^*a*^	2019–2021	*Ae. vexans* (100), *Cq. perturbans* (66.7), *Cx. tarsalis* (18.2), and *Oc. dorsalis* (20)
Partitiviridae	Partitivirus-like *Culex* virus^*a*^	2019–2021	*Cx. tarsalis* (100)
Totiviridae	Gouley virus^*a*^	2020	*Cx. tarsalis* (27.3)
	Snelk virus^*a*^	2019–2021	*Cx. tarsalis* (36.4)
	Hattula totivirus 1	2020	*Oc. triseriatus* (100)
Parvoviridae	Aedes vexans densovirus isolate	2021	*Ae. vexans* (5.3)
	Culex densovirus^*a*^	2020, 2021	*Ae. vexans* (5.3), *Cq. perturbans* (66.7), and *Cx. tarsalis* (9.1)
	Aedes albopictus densovirus	2021	*Cq. perturbans* (33.3) and *Cx. tarsalis* (9.1)
	Grus japonensis parvovirus^*b*^	2021	*Cq. perturbans* (16.7)

^*a*^ Moquito-borne virus previously reported in North America (12, 49, 50, 68–70).

^*b*^ Virus not reported to be naturally vectored by mosquitoes.

^*c*^ Proportion of mosquito cDNA libraries (by species) in which a given virus was detected.

Although viruses dominated the non-host reads, the relative proportions of viral sequences comprising each pooled sample ranged between 0.001% and 2.32%, with the vast majority of <0.2% and an overall average of 0.195% (Table S4). *Ochlerotatus* species tended to have the highest viral percentages, with the four *Oc. dorsalis* sequencing pools ranging between 0.05% and 1.77%, and the only *Oc. flavesce*ns pool showing the highest frequency. On the other hand, the lowest frequency of viral reads was observed in *An. earlei*, which was also represented by only one sequencing pool. The two most prevalent species within the sampling region had mean viral proportions below average: *Ae. vexans* (0.078%) and *Cx. tarsalis* (0.12%).

### Viruses harbored are typically unique to a host species

**Fig 2:**
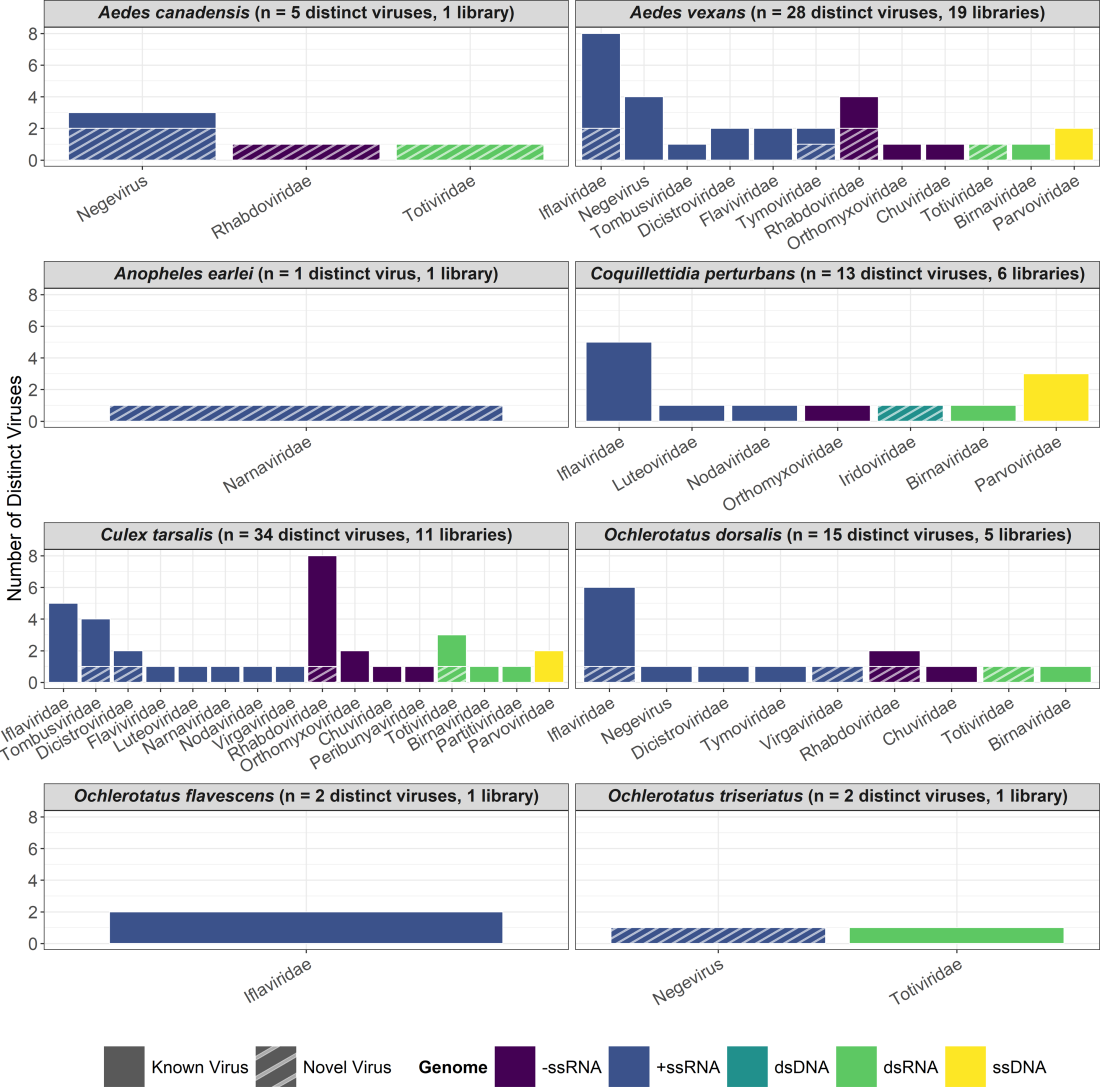
Number of previously reported and novel viruses identified for each mosquito species. Viruses are sorted by family and color coded based on their genome configuration. The number of novel viruses is indicated by hash marks. Also displayed are the total numbers of viruses detected and sequencing libraries for each species.

The proportion of sequencing libraries that each virus was detected in for a given mosquito vector species and year of collection is displayed in Table 1. Some viruses were prevalent in a host species; for instance, Ballard Lake virus was found in all 11 *Ae. vexans* libraries (i.e., sequencing pools) and Merida virus, Astopletus virus, Marma virus, and Placeda virus were identified in each of the 11 *Cx. tarsalis* libraries. Others were detected in low frequencies in the mosquito samples, such as Black queen cell virus and Inari jingmenvirus in *Ae. vexans*. The number of viruses and viral families identified in each mosquito species was largely associated with the number of libraries sequenced (Fig. 2) and, to a lesser extent, the number of sequencing reads generated (Fig. S2). Overall, *Cx. tarsalis* harbored the largest number of previously reported viruses (*n* = 31), followed by *Ae. vexans* (*n* = 23), *C. perturbans* (*n* = 12), and *Oc. dorsalis* (*n* = 11).

**Fig 3:**
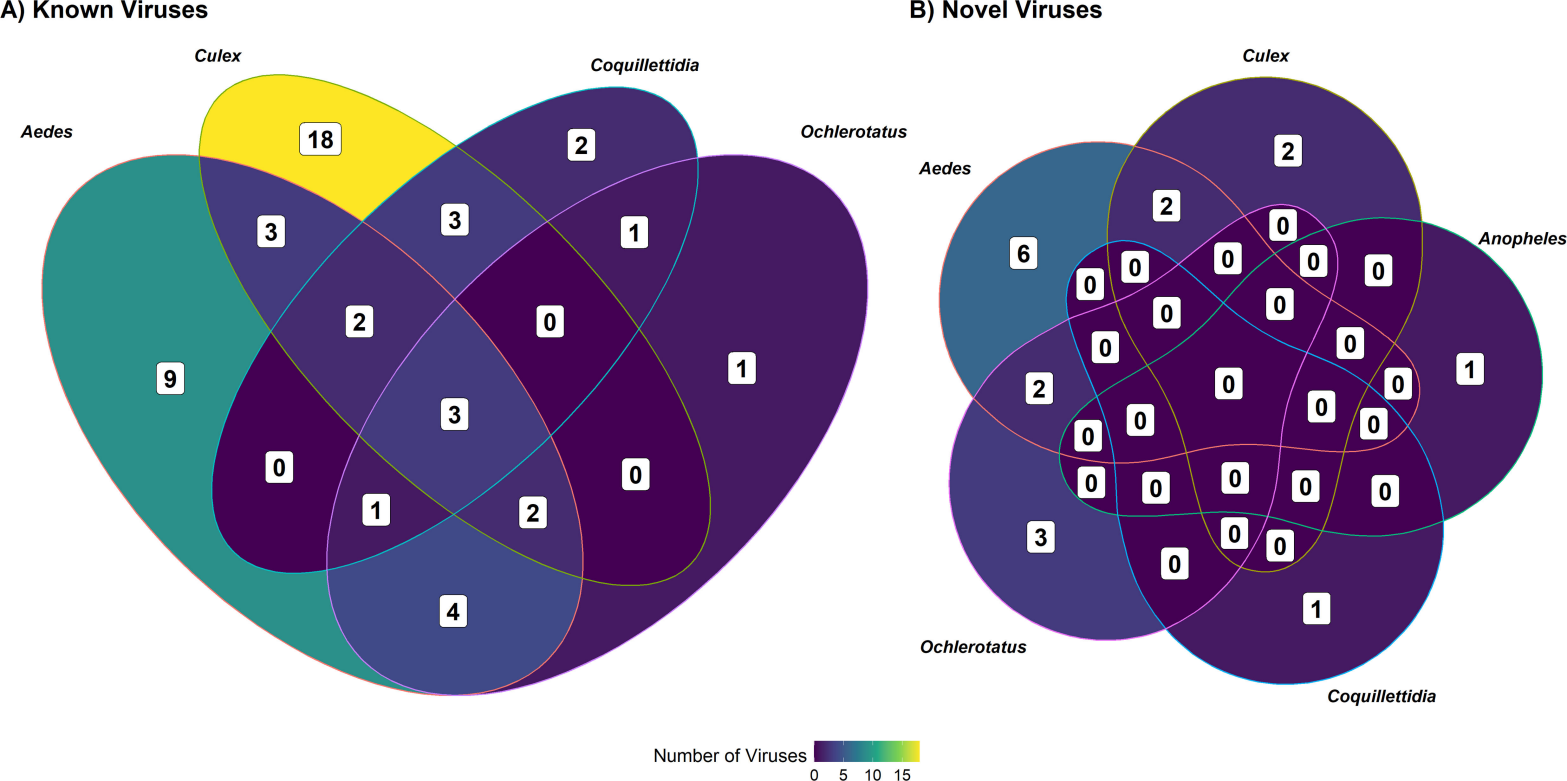
Venn diagram showing the partitioning of (**A**) previously reported and (**B**) novel viruses by mosquito genus. In both cases, the majority of viruses are specific to a given genus.

The partitioning of viruses by mosquito genera is illustrated diagrammatically in Fig. 3A. This Venn diagram emphasizes that viruses are largely unique to a given mosquito genus. Indeed, only 39% (*n* = 19) of viruses were shared among two or more genera, with Hanko iflavirus 1, Pedersore iflavirus, and Ballard Lake virus infecting mosquitoes from all four genera. Moreover, there was virus specificity among species within a genus. Both *Aedes* (*Ae. vexans* and *Ae. canadensis*) and *Ochlerotatus* (*Oc. dorsalis* and *Oc. flavescens*) were represented by two mosquito species, and interestingly, there were no shared viruses identified among species within each of these genera. No known viruses were detected in *An. earlei*, though this species was represented by the second fewest number of sequenced specimens (*n* = 184).

### Novel viruses were identified in most mosquito vector species

In addition to known viruses, we identified a subset of potentially novel viruses whose contig sequences had amino acid similarities of <85% to any organisms currently cataloged in the references databases. We selected this value as the cut-off threshold for the detection of novel viruses, since <90% nucleotide identity is considered a suitable general threshold (53) and we opted to take a conservative approach in our assignment of previously unreported viruses. We also used ortholog prediction to determine the putative viral genome and family of each novel virus. A total of 17 novel viruses were identified infecting Canadian Prairie mosquitoes, which were represented by 1 to 20 contig sequences per virus (Table 2). While the majority (59%) of these novel viruses were +ssRNA, we also detected putatively –ssRNA, dsRNA, and dsDNA viral genomes (Fig. 2). These viruses were classified into 10 different families with Totiviridae, Rhabdoviridae, and Negevirus the most represented at three per family. Four of the viruses (Manitoba picorna-like virus 1, Manitoba tombus-like virus 1, Manitoba Rhabdovirus 1, and Manitoba toti-like virus 1) were detected in all three sampling years. Iridoviridae was the only family where we detected a novel virus but did not identify any previously known viruses. Contig sequences for novel viruses have been deposited in the GenBank database (accession numbers OR448845-OR448861 and OR448915-OR448980).

**TABLE 2:** Family, year(s) reported, sequencing statistics, mosquito species, and Genbank accession numbers for each novel virus

Viral family	Virus	Year(s) reported	No. of contigs	Longest contig (nt)	Coverage depth (mean)	% Identity (median)^*a*^	GenBank accession numbers	Species (% sequencing libraries)^*b*^
Dicistroviridae	Manitoba dicistro-like virus 1	2019	1	7,146	149.27	82.61	OR448845 ^*a*^	*Cx. tarsalis* (9.09)
Iflaviridae	Manitoba iflavirus 1	2020 and 2021	13	2,435	78.09	79.47	OR448846 and OR448915– OR448925	*A^a^e. vexans* (47.37) and *Oc. dorsalis* (20)
Iflaviridae	Manitoba picorna-like virus 1	2019–2021	20	2,114	70.96	83.1	OR448852 and OR448928– OR448946	*Ae. vexans* (63.16)
Narnaviridae	Manitoba narnavirus 1	2021	2	1,760	18.77	80.59	OR448851 and OR448927	*An. earlei* (100)
Negevirus	Manitoba mononega-like virus 1	2021	1	11,180	59.07	73.21	OR448848	*Ae. canadensis* (100)
Negevirus	Manitoba mononega-like virus 2	2021	1	2,477	20.03	75	OR448849	*Ae. canadensis* (100)
Negevirus	Manitoba mononega-like virus 3	2020	1	5,456	42.65	78.57	OR448850	*Oc. triseriatus* (100)
Tombusviridae	Manitoba tombus-like virus 1	2019–2021	5	2,340	157.57	76.19	OR448856 and OR448970– OR448973	*Cx. tarsalis* (45.45)
Tymoviridae	Manitoba tymo-like virus 1	2021	1	6,302	23.67	66.79	OR448860	*Ae. vexans* (5.26)
Virgaviridae	Manitoba virgavirus 1	2020	1	1,675	11.06	83.83	OR448861	*Oc. dorsalis* (20)
Rhabdoviridae	Manitoba Rhabdovirus 1	2019–2021	20	5,085	26.41	77.9	OR448853 and OR448947–OR448965	*Ae. vexans* (73.68) and *Oc. dorsalis* (20)
Rhabdoviridae	Manitoba rhabdovirus 2	2020	1	4,648	51.7	75	OR448854	*Ae. vexans* (5.26)
Rhabdoviridae	Manitoba rhabdovirus 3	2020 and 2021	5	6,071	16.51	84.62	OR448855 and OR448966–OR448969	*Ae. canadensis* (100) and *Cx. tarsalis* (36.36)
Iridoviridae	Manitoba iridescent virus 1	2021	1	797	16.99	78.74	OR448847	*Cq. perturbans* (16.67)
Totiviridae	Manitoba toti-like virus 1	2019–2021	5	8,930	39.82	68.1	OR448857 and OR448974–OR448977	*Ae. vexans* (10.53) and *Cx. tarsalis* (27.27)
Totiviridae	Manitoba toti-like virus 2	2021	1	3,123	13.45	60.24	OR448858	*Ae. canadensis* (100)
Totiviridae	Manitoba toti-like virus 3	2020	4	4,626	15.11	73.5	OR448859 and OR448978–OR448980	*Oc. dorsalis* (80)

^*a*^ All contig sequences for a given virus had amino acid (aa) identities of <85% in the NCBI databases.

^*b*^ Proportion of mosquito cDNA libraries (by species) in which a given virus was detected.

The largest number of novel viruses were identified in *Ae. vexans* (*n* = 6), followed by *Cx. tarsalis*, *Oc. dorsalis*, and *Ae. canadensis* (four per species) (Fig. 3B). Interestingly, both *Ae. canadensis* (4 vs. 1) and *Ae. earlei* (1 vs. 0) were infected with more novel viruses than known viruses. Similar to the known viruses, the novel viruses are largely unique to a given mosquito genus. Only 24% (*n* = 4) of viruses were shared among two or more genera, with no viruses were found across all four genera. Moreover, we did not identify any shared viruses identified among species within a given genus. The only species not infected with a novel virus was *Oc. flavescens*, though this species was represented by a comparatively small number of sequenced specimens (*n* = 270).

### Manitoba viruses were identified globally

Table 3 presents the count of distinct viruses identified in Manitoba, Canada. A total of 66 viruses were identified, comprising 49 viruses previously documented and 17 newly discovered viruses. Additionally, the table links Canada to the countries (if available from the accession) where the closest relatives of each virus were previously identified, based on the highest sequence similarity. For known viruses, the majority were previously reported in the USA (*n* = 28), followed by Finland (*n* = 8), Australia (*n* = 3), and China (*n* = 3). The novel viruses were predominantly associated with viruses found in Finland (*n* = 4), China (*n* = 3), and USA (*n* = 2). Moreover, a significant portion of the viruses previously documented were from North America, including the USA (*n* = 28), Mexico (*n* = 1), and Canada (*n* = 3). The remaining previously reported viruses were documented on different continents: Oceania (*n* = 3), Europe (*n* = 12), Asia (*n* = 6), and South America (*n* = 1). In contrast to known viruses, when considering the closest relatives of the newly discovered viruses, the majority were identified outside of North America. Specifically, Europe (*n* = 7), China (*n* = 5), and Oceania (*n* = 1), while only two had their closest relatives within North America, specifically from the USA.

**TABLE 3:** Geographical distribution of novel and previously reported viruses, based on their closest relative determined through sequence similarity^*a*^

Country	Not novel	Novel
Australia	3	NA
Belgium	1	1
Brazil	1	NA
Canada	3	NA
China	3	3
Finland	8	4
France	1	NA
Hungary	1	1
Mexico	1	NA
Nepal	1	NA
New Zealand	NA	1
Republic of Serbia	NA	1
Russia	1	NA
South Korea	2	1
Sweden	1	1
United States	28	2

^*a*^ NA, not available.

### The non-viral component of the microbiome is significantly smaller

While viruses comprised far and away the largest component of the microbiome for each mosquito species (>99% of non-host sequencing reads), we also detected other sequences of non-host origin (Fig. S3). The majority of the non-host, non-viral reads generated were fungi (53%), most notably Blastocladiomycota, Microsporidia, and Ascomycota. The second most populous group were invertebrate parasites/protozoa (29%), which included Euglenozoa, Apicomplexa, Nematoda, Acari, and Trematoda. The remaining sequences were derived from bacteria (13%),The Viridiplantae (3%), and Chordata (1%). Notable were sequences from the parasitic roundworm *Dirofilaria immitis* (canine heartworm) isolated from two *Ae. vexans* libraries. Moreover, 25.68% of reads derived from protozoa/parasites were from the Apicomplexan genus *Plasmodium*, specifically *Plasmodium gallinaceum* and *Plasmodium relictum* (avian malaria) in *Cx. tarsalis*.

### California serogroup virus screening

To supplement our metatranscriptimic analysis, we carried out targeted screening for California serogroup viruses using a primer pair capable of detecting all viruses of the serogroup viruses (66) and leveraging Sanger sequencing. In 2020, a total of 30 mosquito RNA pools representing 17,423 mosquitoes were screened, and in 2021 we screened 68 pools derived from 16,759 mosquitoes. Only two positive pools were identified, one per year and both from *Ae. vexans*. These pools contained mosquitoes captured in West Manitoba during week 32 and 30 for 2020 and 2021, respectively. Due to an RNA integrity issue, we could only resolve the 2021 positive pool to the serogroup, which was unequivocally identified as Cache Valley virus based on a 251 bp fragment.

## DISCUSSION

The primary objective of our study was to characterize the microbiomes of eight commonly found mosquito species in the Canadian Prairies. More than 35,000 individuals were collected in southern Manitoba over a three-year period (2019 to 2021) and subjected to metatranscriptomic analysis. This approach has been harnessed to catalog the microorganisms harbored by mosquitoes from other geographical regions (12, 47, 50, 71, 72); however, to our knowledge this is the first study done in Canada. A distinct advantage of RNA sequencing over 16S rRNA or shotgun metagenomics is the capability to detect RNA viruses (73). Mosquitoes harbor a diverse range of RNA viruses, many of which can detrimentally affect human health (12, 47). With the exception of *Ae. earlei*, each mosquito species we examined is known to transmit microorganisms of public health concern. Viruses dominated the microbial signature, and included representatives of five types of viral genomes from 19 different families. Similarly, Batson and co-authors showed the microbiome of mosquitoes from the state of California was overwhelmingly composed of viruses (12).

In terms of the virome, several clear trends emerged from our study. As expected, there were strong associations between the number of mosquitoes collected/sequenced and virus discovery. *Aedes vexans* and *Cx. tarsalis* comprised 80% of the specimens and in turn we recovered the greatest number of viruses from those samples. The greater overall sequencing depth and number of libraries and collection sites for both of these species may also contribute (i.e., less false negatives). Indeed, the proportions of sequencing reads of viral origin were below average for sample pools of these species. Nonetheless these are the most ubiquitous species within the sampling region (18, 74, 75), and thus it is conceivable that they naturally harbor the greatest viral diversity. There were some notable exceptions; for instance, *Ae. canadensis* was infected with the same number of novel viruses as *Cx. tarsalis*. This may be attributed to lack of microbiome-related research conducted on this species, as it harbored no previously reported viruses. Another noteworthy pattern was the majority of viruses were unique to a given species, with no observable correlations between phylogenetic distance (i.e., same genera) and viral diversity. This is consistent with the literature, as Batson and colleagues also reported heterogeneity of the virome between species of the same genus (12). As expected, this specificity between virus and host was not discernible at higher taxon ranks; however, as the common viral families (e.g., Iflaviridae, Rhabdoviridae, Negevirus, and Parvoviridae) were represented across several mosquito species.

An aspect of our study of considerable interest was the identification of sequences of viral origin that were not previously reported. The International Committee on Taxonomy of Viruses (ICTV) sets specific standards for virus discovery, with pairwise sequence similarity a primary criterion used. However, many of the viruses we detected are unclassified beyond order or family, making it challenging and somewhat arbitrary to determine a minimum identity threshold to define a new virus. Our amino acid sequence similarity cut-off threshold of 85% for all representative contigs is considered conservative (53), suggesting that we may have underestimated the number of new virus species in our data set. To this end, a limitation for virus discovery from metagenomic or metatranscriptomic analysis is we currently lack bioinformatic tools that can accurately detect viruses exhibiting minimal to no sequence similarity (76). Nonetheless, we identified a total of 17 novel viruses from 11 families, represented by one or multiple contig sequences. In some cases, our assembly algorithms generated a single contig that appears to encompass a nearly complete viral genome. For instance, Manitoba mononega-like virus one has one contig of 11.18 kb, and negeviruses typically have genome sizes between 9 and 10 kb (77). Similarly, Manitoba tymo-like virus 1 had a contig of 6.3 kb (typical genome size of Tymoviridae is 6.0–6.7 kb) (78), and Manitoba dicistro-like virus 1 had a contig of 7.15 kb (typical genome size of Dicistroviridae is 8–10 Kb) (79). As metatranscriptomic studies become increasingly more commonplace, it will be interesting to define the geographical distribution of these viruses and to determine whether they have pathogenicity or potential applications in research.

In addition to the discovery of novel viruses, our study endeavored to detect known viruses, including pathogenic microorganisms of medical importance. West Nile virus, the causal agent of WNV encephalitis, is the primary mosquito-borne pathogen endemic to our sampling region (74). The province of Manitoba has undertaken active surveillance of this virus since 2013, which includes the collection and molecular-based detection of WNV in pools of the regional vector, *Cx. tarsalis*. We did not identify WNV in our mosquito collections, which may be due to relatively low natural circulation of the virus during our sampling years. The province recorded a total of 120 infected *Cx. tarsalis* pools and six human cases between 2019 and 2021, which is well below historical maximums (80). Clinical cases of neuroinvasive disease caused by CSGVs have also been reported in Manitoba (44, 45), and positive mosquito pools have been identified in nearby regions (24). While we did not detect any of these bunyaviruses through RNA sequencing, our more targeted and therefore more sensitive RT-PCR approach identified two positive pools, confirming the presence of Cache Valley virus. This virus was first isolated in 1956 in Cache Valley, Utah, and is considered endemic throughout Canada (81). The virus is most often reported in sheep and was recently shown to have seroprevalence of >33% in individual ewes from farms in Ontario, Canada (82). While NGS is highly sensitive, it is likely that the sequencing depth, coupled with the minimum coverage threshold requirements of our study, was not sufficient to detect low-frequency pathogens. Although no other pathogens of known human or veterinary importance were identified in our study, we did detect viruses belonging to families of public health concern that have not yet been tested for pathogenicity. For instance, we detected Chuvirus, specifically Chuvirus Mos8Chu0, in 90% of *Ae. vexans* libraries and to a lesser extent in *Oc. dorsalis* and *Cx. tarsalis*. Chuvirus Mos8Chu0 was previously reported in *Culiseta minnisotae* from USA (GenBank accession: API61887.1), and the family has been associated with febrile illness in China (83). If this virus does induce disease, it could be of concern, given its broad host range (i.e., detected in three mosquito genera) and ubiquitousness (found in all three sampling years).

Of interest was the detection of Flanders hapavirus (FLAV) in >90% of *Cx. tarsalis* libraries. The virus has no known pathology, but its transmission cycle shares the same avian hosts and *Culex* spp. vectors as WNV (82). Both viruses were shown to co-circulate, with FLAV detectable in *Culex* pools 1–3 weeks prior to peak WNV transmission (67). This suggests that FLAV could act as an early warning system for periods of high WNV transmission. Another bird virus was identified in *Cq.* Flanders hapavirus, *Grus japonensis* parvovirus, though little is known about its transmission cycle (84). Additionally, we detected viruses that are not naturally vectored by mosquitoes, likely occurring due to horizontal transmission through nectar foraging behaviors as evidenced by the diverse plant transcripts found in our data set. Sugar feeding is an important source of nutrients for both sexes, with females ingesting floral and extrafloral nectars throughout their adult life (85, 86). We identified a pathogenic honeybee virus, Black queen cell virus, in *Ae. vexans*, which was recently reported (87). We speculated that the virus was indirectly acquired by mosquitoes foraging at the same nectar sources as honeybees harboring the virus. Three soybean thrip viruses (88) were also identified across multiple mosquito species. One of the viruses, Hubei macula-like virus 3, was previously detected in two mosquito genera, with the co-authors speculating their involvement in a horizontal transmission cycle between arthropods and plants (89). Soybean is ubiquitously cultivated in Manitoba and represents an abundant nectar source, with each plant producing 200–800 flowers and yielding 0.5 µL of nectar per flower (90, 91). To this end, the legume is a preferred nectar source of mosquitoes in the Canadian Prairies (92).

The vast majority of the remaining previously reported viruses identified in our study are likely ISVs; however, it should be emphasized that little to no research has been done on these viruses to assess their pathogenicity or host tropism. Although ISVs infect diverse arthropods, the majority identified thus far have been isolated from mosquitoes (34). Despite their host-range restriction (i.e., only replicate in arthropods), many of the viruses we identified have been found on multiple continents, suggesting they (and perhaps most ISVs) encompass a cosmopolitan distribution. More than half were previously reported in North America, primarily in California, where mosquito metatranscriptomic studies have been concentrated (12, 49, 50). To our knowledge, 17 of the mosquito-borne viruses we detected are newly described in North America and largely infect different species in the same genera. These viruses were primarily discovered not only in *Aedes* and/or *Ochlerotatus* species from Finland (93, 94), Australia (47, 71, 95), and Central Europe (96) but also through meta-analysis (97) and unpublished GenBank deposits. Future studies are needed to determine if these viruses are truly ISVs (i.e., do not infect vertebrates) and their potential application as biomarkers, in biocontrol, and/or disrupting mosquito vectorial capacity.

In addition to viruses, the mosquito microbiome was made up of various fungi, bacteria, protozoa, and invertebrate parasites. Of interest were *P. gallinaceum* and *P. relictum*, which are causal agents of avian influenza. *Plasmodium* parasites that cause avian malaria have been well documented in Manitoba bird populations, infecting birds at rates of upwards of 50% and are present in both migratory and non-migratory birds (98, 99). Given the well-established ornithophilic blood-feeding preferences of *Cx. tarsalis*, this provides a suitable explanation for its mode of transmission from migratory to non-migratory birds. Avian malaria can be detrimental to bird populations that have not yet been exposed to it, which places birds in captivity (e.g., in zoos) and birds in northern regions at elevated risk (100). We also recovered sequences of *D. immitis* in *Ae. vexans*. This roundworm is known to be present in the Manitoba area and is the causative agent of heartworm disease in domestic dogs, cats, and in rare cases humans (101). There were also a variety of transcripts belonging to entomopathogenic fungi (e.g., *Coelomomyces stegomyia*) that could have future application in mosquito control.

Although we identified a wide array of microorganisms, the extent by which viruses dominated the microbial flora of Canadian Prairie mosquitoes is intriguing. To date, most published metatranscriptomic studies have focused on the mosquito virome and excluded the remaining non-host reads. In Batson et al., non-host reads were mapped approximately 84% viral, 8% eukaryotic (including parasites, vertebrate bloodmeals, fungi, and plants), 5% prokaryotic, and 4% taxonomically ambiguous (12). In our study, >99% of non-host reads were of viral origin. This discrepancy does not appear to be an artifact of library preparation, as we applied a poly-A selection stage, but only a small number of the viral families identified were poly-adenylated. A key difference between studies is Batson et al. sequenced individual mosquitoes rather than pooling large numbers of specimens, though it is not apparent how this or other divergent factors (e.g., sampling location and mosquito species) could result in viral enrichment. To this end, it should be emphasized that only a very small percentage of the overall sequencing reads (on average ~0.2%) were of non-host origin. Therefore, relatively small increases in the total number of mapped reads for a given group would result in considerable changes in the overall proportions. As more mosquito metatranscriptomics studies are published, it will be interesting to assess the variability in microbial content that is attributed to viruses.

There are some limitations to our study, which should be briefly addressed. While it is relatively straightforward to distinguish between the species targeted in our study, there is some possibility of misidentification with other morphologically similar species found within our region (e.g., *Aedes striticus* and *Ae. vexans*), leading to nominal contamination within our sequencing pools from species that are not regarded as prominent vector species. Moreover, some of the reads of non-host, non-viral origin could potentially be attributed to surface contamination from extracellular microorganisms rather than mosquito infection. Finally, our study aimed to detect both known and novel microorganisms harbored by mosquitoes, including their prevalence in pooled samples and relative proportions as a product of reads mapping. However, our experimental and sample pooling design was unable to account for several factors that may be of interest, such as the specific months particular arboviruses were detected. Future research is needed to resolve early vs. late season effects of the various microorganisms detected in the Canadian Prairies.

In conclusion, our work builds on the current body of literature characterizing the microbiomes of mosquito species. Advances in metatranscriptomic analysis have allowed for unparalleled resolution into the suite of microorganisms harbored by these hematophagous pests. These studies have taken place on a global scale, though the vast majority of data collected in North America are from the West Coast of the USA. Cataloging the microorganisms infecting mosquitoes provides the baseline information needed for more targeted studies aimed at elucidating how the microbiome influences development, longevity, immunity, and vector competence. We demonstrated that the virome is rich in diversity and represents the largest component of the microbiome, consisting of pathogens, ISVs, and even non-mosquito-borne viruses. We report on several new viruses, and as metatranscriptomics becomes more pervasive, a nearly exhaustive list of novel viruses should emerge. Future studies should explore their human and veterinary implications, interactions with other arboviruses, temporal relationships, and rates of co-infection.

## Data Availability

The raw sequence reads can be retrieved from the National Center for Biotechnology Information Short Sequence Read Archive (SRA) under the SRA accession number PRJNA793247. The contig sequences for each novel virus have been deposited in the GenBank database (accession numbers OR448845-OR448861 and OR448915-OR448980).

## SUPPLEMENTAL MATERIAL

Supplemental Figures msphere.00203-24-s0001.docx Figures S1 to S3.

Supplemental Tables msphere.00203-24-s0002.docx Tables S1 to S4.

ASM does not own the copyrights to Supplemental Material that may be linked to, or accessed through, an article. The authors have granted ASM a non-exclusive, world-wide license to publish the Supplemental Material files. Please contact the corresponding author directly for reuse.
