# Supplementary material for: Metatranscriptomic analysis of common mosquito vector species in the Canadian Prairies
Source: mSphere. 2024 Jun 24;9(7):e00203-24. doi: 10.1128/msphere.00203-24 (PMC11288045; doi:10.1128/msphere.00203-24)

**Supplementary Figure S1**. Metadata statistics for each sequencing library (horizontal line). Each library consists of mosquitoes pooled by year, location, and species. Libraries are represented by three icons; total number of reads (left), number of reads after quality filtering (middle), and number reads non-host reads (right). Also labelled for each library is the number of mosquito specimens comprising each RNA pool.


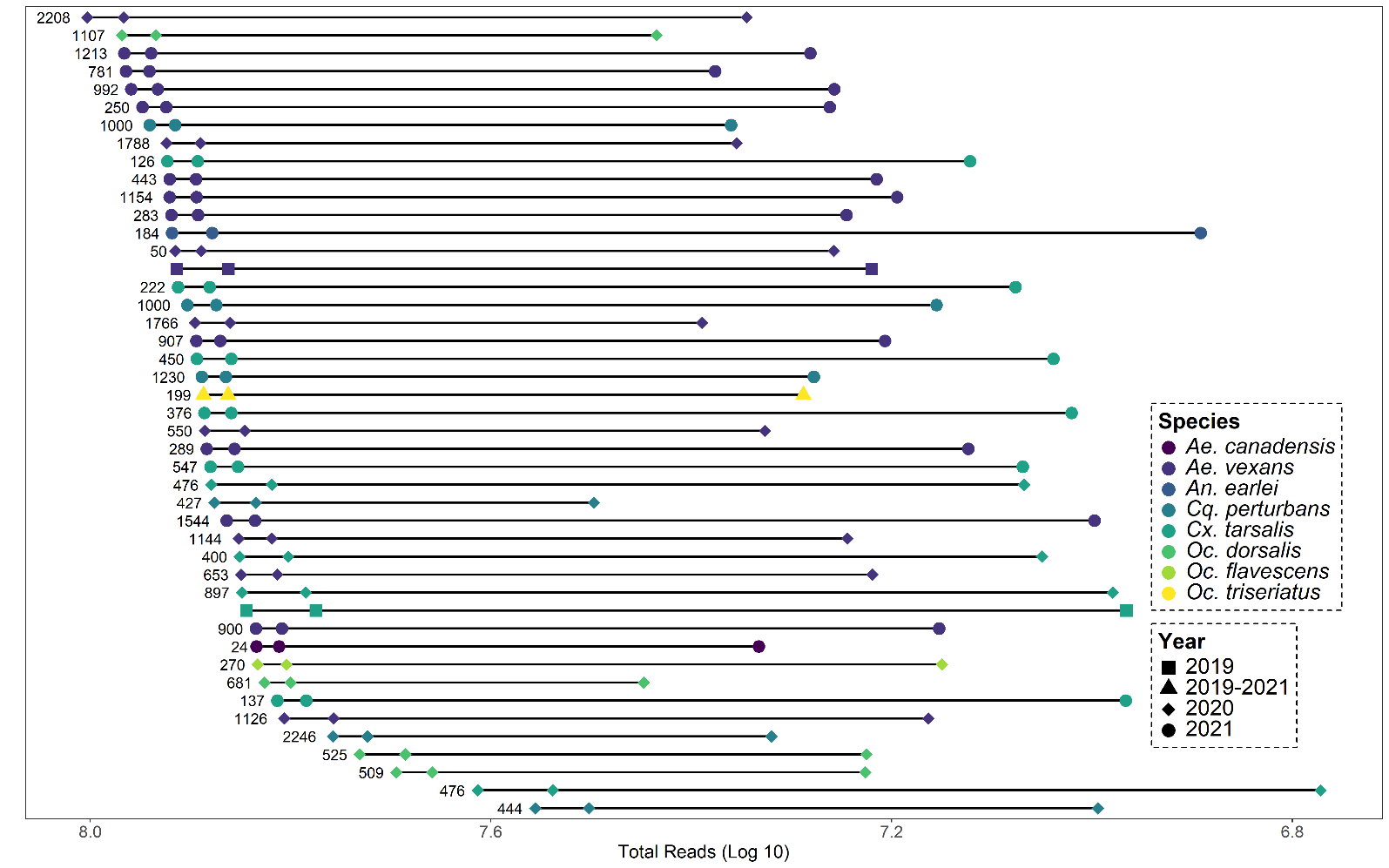


**Supplementary Figure S2**. Total number of viral reads from previously reported and novel viruses identified in each mosquito species. Viruses are sorted by family and colour coded based on their genome configuration. Also displayed is the number of sequencing libraries for each species.

**
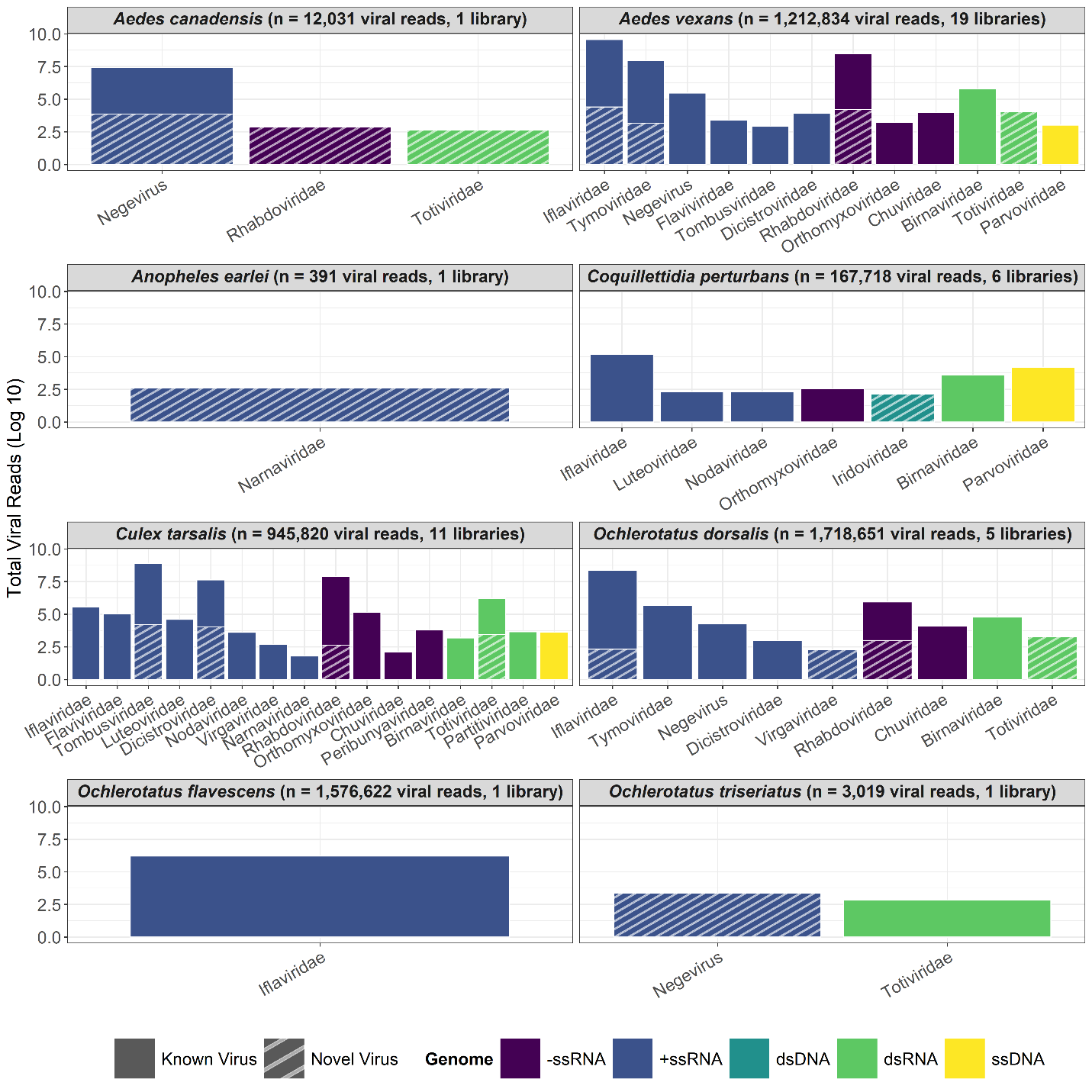
**

**Supplementary Figure S3**. Non-viral, non-host organisms identified in Canadian Prairie mosquitoes. Displayed is the (A) number of total sequencing reads (B) and number of organisms detected for the various groups of fungi, invertebrate parasites/protozoa, plants, and vertebrates. The bars are colour-coded based on the mosquito species each organism was identified in.

**A)**
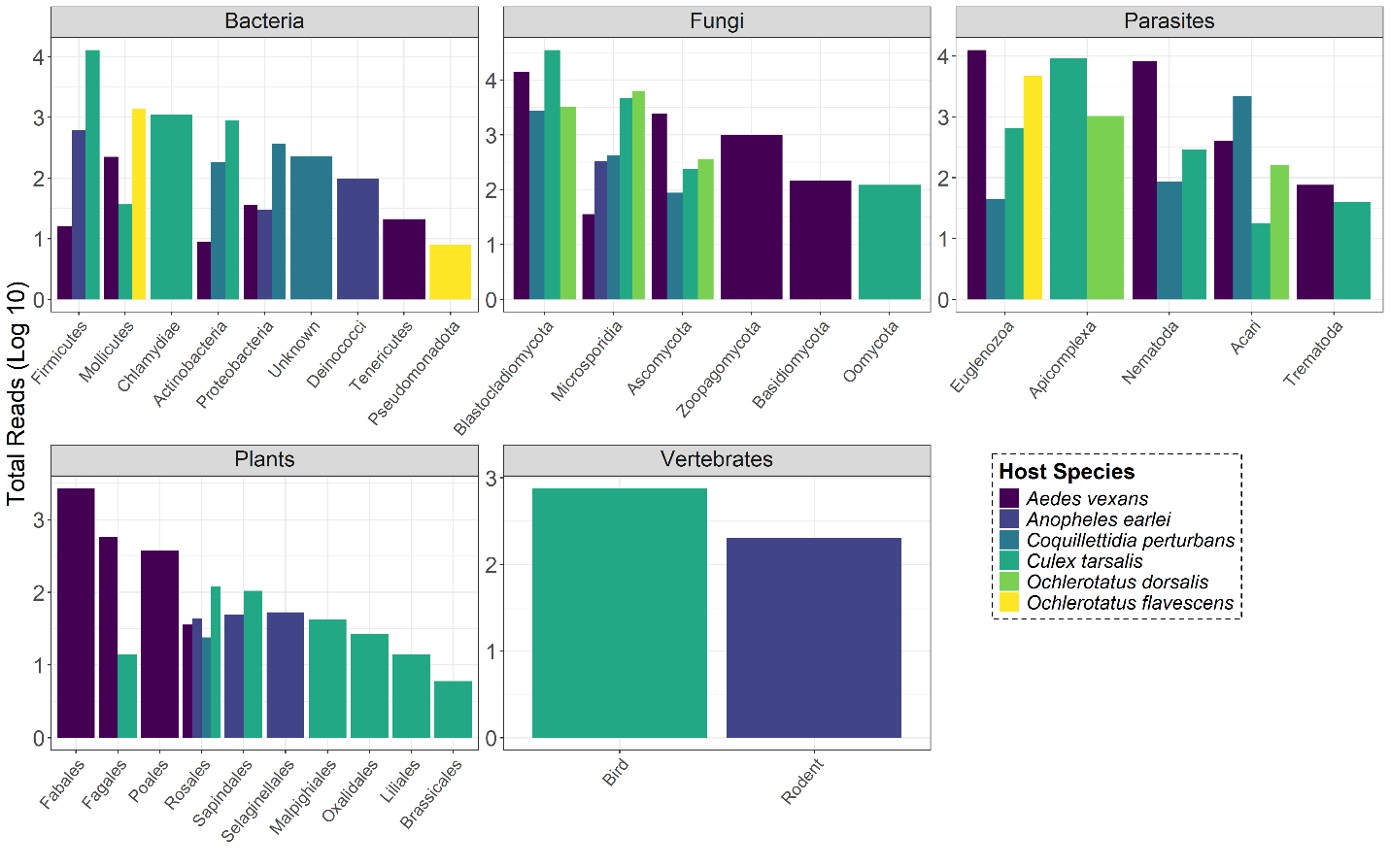


**B)**


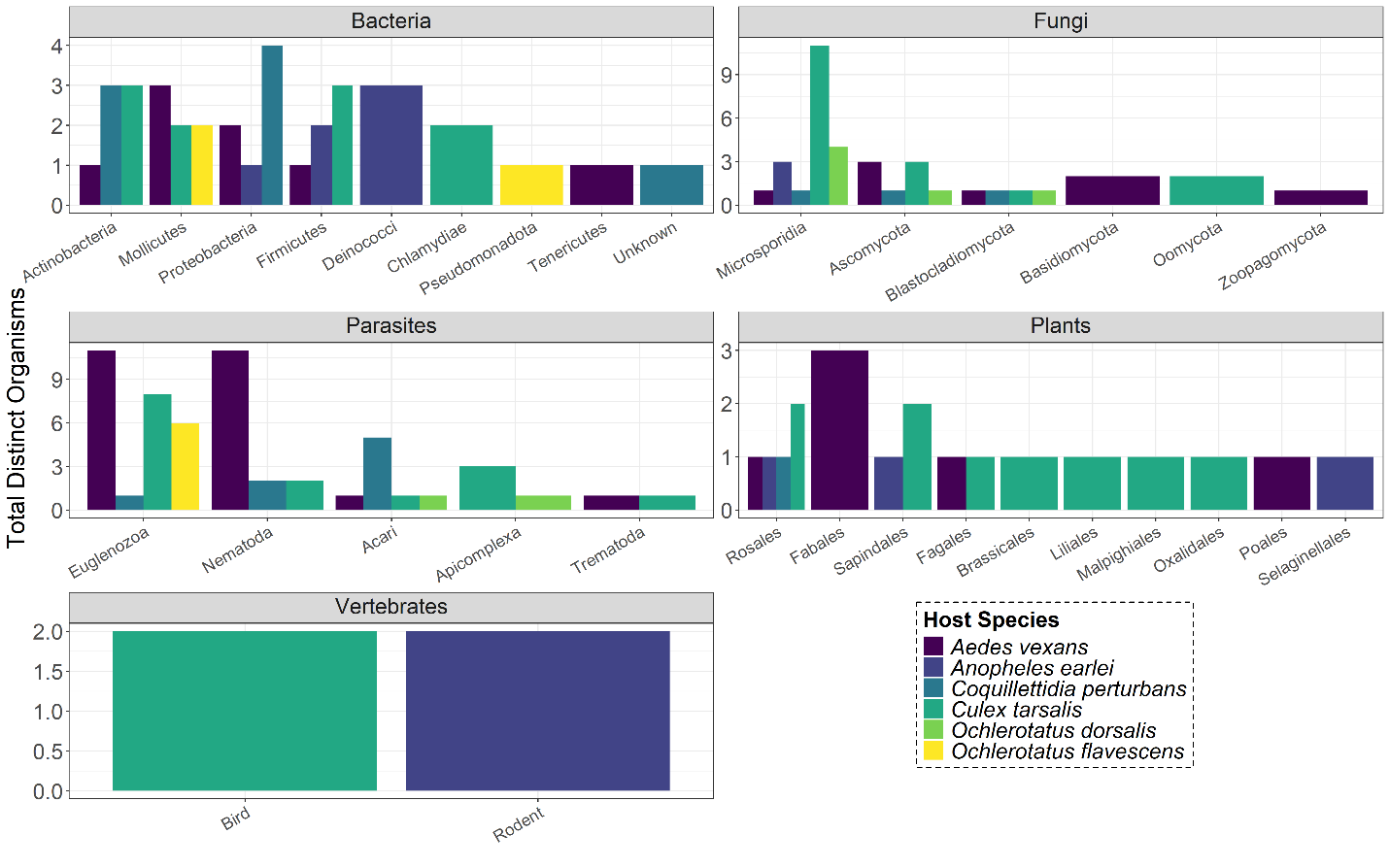

Supplement: Supplemental Figures — Figures S1 to S3. [file msphere.00203-24-s0001.docx]
