# Supplementary material for: Metatranscriptomic analysis of common mosquito vector species in the Canadian Prairies
Source: mSphere. 2024 Jun 24;9(7):e00203-24. doi: 10.1128/msphere.00203-24 (PMC11288045; doi:10.1128/msphere.00203-24)
Supplement: Supplemental Tables — Tables S1 to S4. [file msphere.00203-24-s0002.docx]

**Supplementary Table S1**. Description of the sampling sites mosquitoes were collected from. This includes the city/town, region, community, GPS coordinates, and a general description of the sampling site.

| City/town | Region | Community | Coordinates | Description |
| --- | --- | --- | --- | --- |
| Virden | West | Rural | 49.848668; -100.93253 | Adjacent a forest and horse riding area |
| Brandon-A | West | Urban | 49.823684, -99.962229 | Backyard in a residential area |
| Brandon-B | West | Urban | 49.831754, -99.966649 | Backyard in a residential area with some trees situated nearby |
| Brandon-C | West | Urban | 49.843075, -99.933283 | Backyard in a residential area, prominent gardens in this area |
| Cypress River | West | Rural | 49.556221; -99.090882 | Situated at forest edge, adjacent a swamp and field |
| Shoal Lake | West | Rural | 50.438087; -100.59073 | Next to a small lake and human dwellings |
| Souris | West | Rural | 49.620799; -100.25830 | Forest edge, close to residential area |
| Carberry | West | Rural | 49.867704; -99.360176 | Situated adjacent a forest and field |
| Killarney | West | Rural | 49.183215; -99.664190 | Close to town center, overlooking a field |
| Altona | East | Rural | 49.103498; -97.555495 | Field near the forest edge |
| Steinbach | East | Rural | 49.528528; -96.691092 | Forest edge, near a field with human dwelling in the distance |
| Boissevain | East | Rural | 49.133000; -100.03108 | Adjacent human dwelling and forested area |
| Virden | East | Rural | 49.850833;-100.931666 | Residential area, field and sparse forest nearby |
| Eastern MB | East | Urban | East of -98.29263 | Various residential sites in Winnipeg, Steinbach and West St. Paul |

**Supplementary Table S2**. Information pertaining to mosquito collection. Included for each specimen pool is the year collected, sampling locations, mosquito species, number of RNA extractions, months samples were collected, sample type, RNA concentration and quality indices, number of sequencing reads and the number of reads passing quality control (QC) and filtered.

**Supplementary Table S3.** Sequencing statistics for the 40 previously reported (i.e., known) viruses detected in our mosquito pools.

| **Virus** | **Contigs** | **Longest Contig (nt)** | **Reads** | **Coverage Depth** | | | **aa Percent Identity** | | | |
| --- | --- | --- | --- | --- | --- | --- | --- | --- | --- | --- |
|  |  |  |  | **Mean** | **Min** | **Max** | **Mean** | **Min** | **Max** | **Median** |
| **+ssRNA - Dicistroviridae** | | | | | | | | | | |
| Black queen cell virus | 2 | 6035 | 1,613 | 17 | 11.15 | 22.85 | 100 | 100 | 100 | 100 |
| Soybean thrips dicistrovirus | 11 | 9121 | 11,192 | 17.32 | 10.11 | 29.01 | 100 | 100 | 100 | 100 |
| **+ssRNA - Flaviviridae** | | | | | | | | | | |
| Inari jingmenvirus | 1 | 1267 | 139 | 11.02 | 11.02 | 11.02 | 100 | 100 | 100 | 100 |
| Placeda virus | 169 | 11737 | 106,682 | 90.37 | 10.02 | 1,114.82 | 97.87 | 86.24 | 100 | 98.55 |
| **+ssRNA - Iflaviridae** | | | | | | | | | | |
| Cafluga virus | 2 | 3426 | 976 | 20.82 | 19.41 | 22.24 | 99.77 | 99.55 | 100 | 99.77 |
| Culex Iflavi-like virus 4 | 23 | 9811 | 13,815 | 22.81 | 10.65 | 58.9 | 99.81 | 96.2 | 100 | 100 |
| Culex iflavilike virus 3 | 183 | 1824 | 266,451 | 284.65 | 18.65 | 1,269.06 | 99.49 | 94.19 | 100 | 100 |
| Hanko iflavirus 1 | 30 | 9246 | 2,938,402 | 3,394.01 | 10.99 | 19,795.35 | 97.49 | 93.45 | 100 | 97.3 |
| Hanko iflavirus 2 | 16 | 9206 | 19,456 | 93.72 | 18.5 | 208.48 | 97.05 | 89.58 | 100 | 100 |
| Hubei arthropod virus 1 | 1 | 2967 | 352 | 11.95 | 11.95 | 11.95 | 100 | 100 | 100 | 100 |
| Pedersore iflavirus | 25 | 9899 | 39,865 | 28.41 | 10.35 | 266.71 | 93.47 | 89.14 | 100 | 93.38 |
| Soybean thrips iflavirus 4 | 1 | 3835 | 636 | 16.6 | 16.6 | 16.6 | 100 | 100 | 100 | 100 |
| Thrace picorna-like virus 1 | 2 | 1172 | 2,010 | 82.45 | 34.1 | 130.79 | 91.62 | 89.58 | 93.65 | 91.62 |
| Yongsan picorna-like virus 1 | 64 | 4085 | 55,324 | 62.58 | 10.27 | 180.88 | 89.4 | 85.05 | 100 | 89.11 |
| Yongsan picorna-like virus 2 | 4 | 9573 | 58,132 | 165.26 | 34.98 | 535.91 | 100 | 100 | 100 | 100 |
| **+ssRNA - Luteoviridae** | | | | | | | | | | |
| Marma virus | 23 | 3160 | 39,344 | 70.95 | 11.62 | 129.64 | 100 | 100 | 100 | 100 |
| **+ssRNA - Narnaviridae** | | | | | | | | | | |
| Culex narnavirus 1 | 1 | 513 | 63 | 12.07 | 12.07 | 12.07 | 100 | 100 | 100 | 100 |
| **+ssRNA - Negevirus** | | | | | | | | | | |
| Big Cypress virus | 3 | 9570 | 6,079 | 37.12 | 18.89 | 65.64 | 98.77 | 96.3 | 100 | 100 |
| Bro virus | 1 | 11375 | 3,826 | 33.85 | 33.85 | 33.85 | 90 | 90 | 90 | 90 |
| Cordoba virus | 14 | 5133 | 135,594 | 548.17 | 17.51 | 2,225.85 | 97.1 | 93.75 | 100 | 97.23 |
| Mekrijarvi Negevirus | 6 | 9873 | 167,549 | 294.01 | 30.28 | 1,262.63 | 94.13 | 90.78 | 100 | 93.4 |
| Utsjoki negevirus 3 | 2 | 989 | 416 | 21.12 | 18 | 24.24 | 99.02 | 99.01 | 99.03 | 99.02 |
| **+ssRNA - Nodaviridae** | | | | | | | | | | |
| Hubei noda-like virus 12 | 2 | 4762 | 4,244 | 54.47 | 23.6 | 85.34 | 95.83 | 91.67 | 100 | 95.83 |
| **+ssRNA - Tombusviridae** | | | | | | | | | | |
| Des Moines River virus | 4 | 2361 | 35,117 | 486.06 | 34.94 | 897.17 | 100 | 100 | 100 | 100 |
| Hubei mosquito virus 4 | 6 | 5103 | 2,919 | 16.68 | 10.02 | 39.59 | 96.5 | 92.65 | 100 | 95.7 |
| Tiger mosquito bi-segmented tombus-like virus | 1 | 2351 | 13,049 | 557.41 | 557.41 | 557.41 | 100 | 100 | 100 | 100 |
| **+ssRNA - Tymoviridae** | | | | | | | | | | |
| Hubei macula-like virus 3 | 8 | 6059 | 530,385 | 1,092.52 | 11.15 | 7,749.86 | 96.13 | 92.15 | 100 | 96.63 |
| **+ssRNA - Virgaviridae** | | | | | | | | | | |
| Hubei virga-like virus 2 | 6 | 947 | 505 | 13.26 | 10.94 | 15.5 | 99.04 | 96.27 | 100 | 100 |
| **-ssRNA - Chuviridae** | | | | | | | | | | |
| Chuvirus | 39 | 6799 | 22,130 | 28 | 10.17 | 214.52 | 97.49 | 89.66 | 100 | 97.02 |
| **-ssRNA - Orthomyxoviridae** | | | | | | | | | | |
| Astopletus virus | 29 | 1044 | 5,975 | 32.63 | 10.91 | 107.95 | 99.06 | 95.77 | 100 | 100 |
| Wuhan mosquito virus 6 | 101 | 2467 | 133,667 | 176.35 | 12.58 | 641.58 | 99.76 | 97.69 | 100 | 100 |
| **-ssRNA - Peribunyaviridae** | | | | | | | | | | |
| Culex bunyavirus 2 | 19 | 1896 | 6,255 | 23.92 | 10.28 | 49.84 | 100 | 100 | 100 | 100 |
| **-ssRNA - Rhabdoviridae** | | | | | | | | | | |
| Canya virus | 9 | 2444 | 2,613 | 29.27 | 11.64 | 66.33 | 90.87 | 85.05 | 100 | 85.92 |
| Culex Rhabdo-like virus | 4 | 1973 | 807 | 14.68 | 10.87 | 23.28 | 100 | 100 | 100 | 100 |
| Culex rhabdovirus | 2 | 429 | 997 | 116.26 | 74.47 | 158.06 | 100 | 100 | 100 | 100 |
| Elisy virus | 21 | 1817 | 3,849 | 19.29 | 10.1 | 40.69 | 99.27 | 96.41 | 100 | 100 |
| Flanders hapavirus | 81 | 8773 | 104,235 | 113.05 | 10 | 686.87 | 99.9 | 95.96 | 100 | 100 |
| Manitoba virus | 5 | 1589 | 1,003 | 19.71 | 15.74 | 24.8 | 99.76 | 98.8 | 100 | 100 |
| Merida virus | 55 | 7483 | 76,697 | 91.6 | 11.06 | 276.87 | 99.86 | 97.14 | 100 | 100 |
| Riverside virus 1 | 17 | 8033 | 18,623 | 45.08 | 10.8 | 120.41 | 90.2 | 85.32 | 93.01 | 90 |
| **dsRNA - Birnaviridae** | | | | | | | | | | |
| Ballard Lake virus | 48 | 3472 | 682,541 | 431.81 | 10.84 | 2,260.59 | 99.86 | 93.27 | 100 | 100 |
| **dsRNA - Partitiviridae** | | | | | | | | | | |
| Partitivirus-like Culex mosquito virus | 12 | 1751 | 4,477 | 23.28 | 11.49 | 45.83 | 100 | 100 | 100 | 100 |
| **dsRNA - Totiviridae** | | | | | | | | | | |
| Gouley virus | 3 | 368 | 117 | 12.22 | 10.42 | 14.89 | 95.04 | 92.86 | 97.67 | 94.59 |
| Hattula totivirus 1 | 1 | 4909 | 702 | 14.41 | 14.41 | 14.41 | 89.07 | 89.07 | 89.07 | 89.07 |
| Snelk virus | 4 | 295 | 496 | 41.15 | 23.15 | 67.61 | 100 | 100 | 100 | 100 |
| **ssDNA - Parvoviridae** | | | | | | | | | | |
| Aedes albopictus densovirus | 4 | 3355 | 14,051 | 139.82 | 94.27 | 203.18 | 97.46 | 89.86 | 100 | 100 |
| Aedes vexans densovirus isolate | 1 | 3367 | 594 | 17.69 | 17.69 | 17.69 | 100 | 100 | 100 | 100 |
| Culex densovirus | 8 | 1697 | 6,035 | 127.22 | 11.65 | 490.03 | 99.56 | 97.71 | 100 | 100 |
| Grus japonensis parvoviridae | 1 | 570 | 366 | 60.82 | 60.82 | 60.82 | 100 | 100 | 100 | 100 |
|  |  |  |  |  |  |  |  |  |  |  |

**Supplementary Table S4.** The number and relative proportions of sequencing reads of viral origin for each mosquito pool. Displayed is the location, number of mosquitoes collected, mosquito species, and collection year for each pooled sequencing library.
